# Supplementary material for: State social distancing restrictions and nursing home outcomes
Source: Sci Rep. 2022 Jan 20;12:1058. doi: 10.1038/s41598-022-05011-6 (PMC8776882; doi:10.1038/s41598-022-05011-6)
Supplement: Supplementary file 1 — Supplementary Tables. [file 41598_2022_5011_MOESM1_ESM.docx]

**Supplements to “State social distancing restrictions and nursing home outcomes”**

Yue Li, PhD ^1^

Zijing Cheng, MS ^1^

Xueya Cai, PhD ^2^

Yunjiao Mao, MS ^1^

Helena Temkin-Greener, PhD ^1^

^1^ Department of Public Health Sciences, Division of Health Policy and Outcomes Research, University of Rochester Medical Center.

^2^ Department of Biostatistics and Computational Biology, University of Rochester Medical Center.

**Supplementary Table S1**. Nursing home characteristics by nursing home quartile groups of proportion of racial/ethnic minority residents

| **Nursing home characteristic** | All nursing homes, counties, or states | Nursing homes by proportion of racial/ethnic minority residents* | | | |
| --- | --- | --- | --- | --- | --- |
|  |  | Low | Medium | Medium-high | High |
|  | Mean±SD or N (%) | | | | |
| Number of nursing homes | 13,581 | 3,390  (24.96) | 3,417  (25.16) | 3,385  (24.92) | 3,389  (24.95) |
| Weekly number of new Covid-19 confirmed cases among residents^a^ | 0.59±2.8 | 0.31±2.12 | 0.48±2.40 | 0.80±3.42 | 0.79±2.03 |
| 0 | 11,748  (86.50) | 3,153  (93.01) | 3,037  (88.88) | 2,846  (84.08) | 2,712  (80.02) |
| 1-10 | 1,482  (10.91) | 187  (5.52) | 319  (9.34) | 417  (12.32) | 559  (16.49) |
| >10 | 351  (2.58) | 50  (1.47) | 61  (1.78) | 122  (3.60) | 118  (3.48) |
| Weekly number of new Covid-19 confirmed cases among staff^a^ | 0.51±1.63 | 0.34±1.39 | 0.41±1.33 | 0.66±1.86 | 0.62±1.79 |
| 0 | 10,621  (78.20) | 2,869  (84.63) | 2,774  (81.18) | 2,505  (74.00) | 2,473  (72.97) |
| 1-10 | 2,745  (20.21) | 485  (14.31) | 611  (17.88) | 811  (23.96) | 838  (24.73) |
| >10 | 215  (1.58) | 36  (1.06) | 32  (0.93) | 69  (2.04) | 78  (2.30) |
| Weekly number of new Covid-19 related deaths among residents^b^ | 0.10±0.55 | 0.06±0.47 | 0.08±0.54 | 0.13±0.58 | 0.13±0.60 |
| 0 | 12,693  (93.46) | 3,249  (95.84) | 3,248  (95.05) | 3,102  (91.64) | 3,094  (91.30) |
| 1-5 | 725  (5.34) | 117  (3.45) | 141  (4.13) | 234  (6.91) | 233  (6.88) |
| >5 | 163  (1.20) | 24  (0.71) | 28  (0.82) | 49  (1.45) | 62  (1.83) |
| Weekly number of new non-Covid-19 related deaths among residents^b^ | 0.41±1.84 | 0.47±2.11 | 0.45±1.97 | 0.44±2.09 | 0.32±1.11 |
| 0 | 9,984  (73.52) | 2,449  (72.24) | 2,455  (71.87) | 2,489  (73.53) | 2,591  (76.45) |
| 1-5 | 3,409  (25.10) | 905  (26.70) | 929  (27.20) | 839  (24.79) | 736  (21.72) |
| >5 | 187  (1.38) | 36  (1.06) | 32  (0.94) | 57  (1.68) | 62  (1.83) |
| Total number of certified beds | 106.24±55.86 | 86.26±44.67 | 103.32±53.46 | 116.44±56.46 | 124.40±58.46 |
| Number of residents | 85.69±48.78 | 70.04±39.74 | 82.57±46.57 | 92.86±49.10 | 101.65±50.69 |
| Ownership |  |  |  |  |  |
| For-profit | 9,576  (70.51) | 1,712  (50.50) | 2,347  (68.69) | 2,643  (78.08) | 2,874  (84.80) |
| Non-profit | 3,150  (23.19) | 1,368  (40.35) | 851  (24.90) | 563  (16.63) | 368  (10.86) |
| Government owned | 855  (6.30) | 310  (9.14) | 219  (6.41) | 179  (5.29) | 147  (4.34) |
| Chain affiliated | 8,045  (59.24) | 1,757  (51.83) | 2,065  (60.43) | 2,121  (62.66) | 2,102  (62.02) |
| Hospital affiliated | 427  (3.14) | 182  (5.37) | 97  (2.84) | 66  (1.95) | 82  (2.42) |
| Percentage of Medicaid residents, % | 59.84±23.08 | 52.02±22.50 | 56.95±22.18 | 62.82±20.44 | 69.33±19.89 |
| Percentage of Medicare residents, % | 13.80±13.31 | 12.60±12.20 | 14.58±13.09 | 14.24±12.39 | 11.81±9.80 |
| Case mix index score | 1.29±0.16 | 1.27±0.11 | 1.29±0.12 | 1.29±0.14 | 1.31±0.21 |
| RN hours per resident day | 0.67±0.48 | 0.76±0.41 | 0.68±0.37 | 0.60±0.37 | 0.52±0.35 |
| Total nurse hours per resident day | 3.83±0.88 | 3.95±0.83 | 3.82±0.77 | 3.71±0.74 | 3.69±0.80 |
| Overall five-star rating | 3.19±1.41 | 3.64±1.32 | 3.31±1.37 | 2.99±1.39 | 2.76±1.39 |
| Cumulative number of Covid-19 confirmed cases among residents before August 11^c^ | 2.68±8.81 | 1.07±4.96 | 1.89±7.17 | 3.08±9.48 | 4.74±11.87 |
| Cumulative number of Covid-19 confirmed cases among staff before August 11^c^ | 2.46±7.11 | 1.15±3.63 | 1.99±5.29 | 2.88±10.00 | 3.86±7.72 |
| Cumulative number of Covid-19 deaths among residents before August 11^c^ | 0.76±2.82 | 0.38±1.97 | 0.65±2.78 | 0.84±2.76 | 1.16±3.44 |
| **County characteristic** |  |  |  |  |  |
| Cumulative number of Covid-19 cases before August 11, x1k^c^ | 3.67±12.47 | 0.76±4.02 | 1.39±5.71 | 2.88±9.31 | 8.57±19.58 |
| Cumulative number of Covid-19 deaths before August 11, x1k^c^ | 0.09±0.26 | 0.03±0.12 | 0.05±0.14 | 0.08±0.20 | 0.20±0.40 |
| Total populationx100k | 8.25±17.94 | 2.41±6.43 | 4.01±7.99 | 7.23±13.30 | 17.49±27.29 |
| **State characteristic** |  |  |  |  |  |
| Cumulative rate of Covid-19 cases before August 11 (per one thousand)^c^ | 3.56±3.11 | 2.96±2.37 | 3.20±2.85 | 3.76±3.38 | 4.26±3.52 |
| Cumulative rate of Covid-19 deaths before August 11 (per one thousand)^c^ | 0.10±0.08 | 0.11±0.08 | 0.11±0.09 | 0.10±0.09 | 0.10±0.08 |
| Percentage population ≥65 years, % | 14.86±2.00 | 15.38±1.61 | 15.08±1.87 | 14.73±2.18 | 14.34±2.13 |
| Percentage of non-white population, % | 30.40±10.78 | 24.43±9.46 | 27.43±9.43 | 31.75±9.38 | 37.09±9.76 |

* All p-values were <0.001 for comparisons of group differences based on analyses of variance for continuous variables, and chi-square tests for categorical variables.

^a^ Numbers are for the reporting week ending on August 23 (Monday August 17 to Sunday August 23).

^b^ Numbers are for the reporting week ending on September 6 (Monday August 31 to Sunday September 6).

^c^ Cumulative numbers are reported for the period from May 25 to August 9, 2020.

SD=standard deviation; RN=registered nurse.

**Table S2**. Associations between the strength of state social distancing measures and nursing home COVID-19 and non-COVID-19 outcomes – **sensitivity analyses in which rankings of state social distancing measures are a continuous variable**.^a^

| **Two-part model for confirmed COVID-19 cases among residents** | **β-coefficient** | **OR or IRR^b^**  **(95% CI)** | **P-value** |
| --- | --- | --- | --- |
| **Part 1: likelihood of ≥1 case** |  |  |  |
| Ranking of state social distancing measures x 10 | -0.05 | 0.95 (0.93-0.98) | <0.001 |
| **Part 2: count of cases conditional on ≥1 case** |  |  |  |
| Ranking of state social distancing measures x 10 | -0.02 | 0.98 (0.96-1.01) | 0.218 |
|  |  |  |  |
| **Two-part model for confirmed COVID-19 cases among staff** |  |  |  |
| **Part 1: likelihood of ≥1 case** |  |  |  |
| Ranking of state social distancing measures x 10 | -0.07 | 0.93 (0.91-0.95) | <0.001 |
| **Part 2: count of cases conditional on ≥1 case** |  |  |  |
| Ranking of state social distancing measures x 10 | -0.02 | 0.98 (0.96-1.00) | 0.051 |
|  |  |  |  |
| **Two-part model for COVID-19 related deaths among residents** |  |  |  |
| **Part 1: likelihood of ≥1 death** |  |  |  |
| Ranking of state social distancing measures x 10 | -0.09 | 0.92 (0.89-0.95) | <0.001 |
| **Part 2: count of deaths conditional on ≥1 death** |  |  |  |
| Ranking of state social distancing measures x 10 | 0.02 | 1.02 (1.00-1.04) | 0.123 |
|  |  |  |  |
| **Two-part model for non-COVID-19 deaths among residents** |  |  |  |
| **Part 1: likelihood of ≥1 death** |  |  |  |
| Ranking of state social distancing measures x 10 | 0.004 | 1.00 (0.99-1.02) | 0.665 |
| **Part 2: count of deaths conditional on ≥1 death** |  |  |  |
| Ranking of state social distancing measures x 10 | 0.03 | 1.03 (0.99-1.07) | 0.119 |
|  |  |  |  |

^a^ Based on two-part models for confirmed cases and deaths separately that adjusted for nursing home, county, and state covariates, time trend, and the clustering of repeated observations of nursing homes.

^b^ ORs are reported for part 1 of the two-part models and IRR are reported for part 2 of the two-part models.

OR=odds ratio; IRR=incidence rate ratio; 95% CI=95% confidence interval.

**Table S3**. Associations between the strength of state social distancing measures and nursing home COVID-19 and non-COVID-19 outcomes – **sensitivity analyses in which rankings of state social distancing measures are categorized as tertile groups**.^a^

| **Two-part model for confirmed COVID-19 cases among residents** | **β-coefficient** | **OR or IRR^b^**  **(95% CI)** | **P-value** |
| --- | --- | --- | --- |
| **Part 1: likelihood of ≥1 case** |  |  |  |
| 2^nd^ tertile in strength of state social distancing measures^c^ | -0.06 | 0.94 (0.87-1.02) | 0.122 |
| 3^rd^ tertile in strength of state social distancing measures^c^ | -0.13 | 0.88 (0.82-1.05) | 0.003 |
| **Part 2: count of cases conditional on ≥1 case** |  |  |  |
| 2^nd^ tertile in strength of state social distancing measures^c^ | -0.10 | 0.91 (0.83-0.99) | 0.031 |
| 3^rd^ tertile in strength of state social distancing measures^c^ | -0.06 | 0.94 (0.86-1.03) | 0.176 |
|  |  |  |  |
| **Two-part model for confirmed COVID-19 cases among staff** |  |  |  |
| **Part 1: likelihood of ≥1 case** |  |  |  |
| 2^nd^ tertile in strength of state social distancing measures^c^ | -0.07 | 0.93 (0.88-0.99) | 0.023 |
| 3^rd^ tertile in strength of state social distancing measures^c^ | -0.24 | 0.79 (0.74-0.84) | <0.001 |
| **Part 2: count of cases conditional on ≥1 case** |  |  |  |
| 2^nd^ tertile in strength of state social distancing measures^c^ | -0.01 | 1.00 (0.93-1.06) | 0.880 |
| 3^rd^ tertile in strength of state social distancing measures^c^ | -0.06 | 0.94 (0.88-1.00) | 0.052 |
|  |  |  |  |
| **Two-part model for COVID-19 related deaths among residents** |  |  |  |
| **Part 1: likelihood of ≥1 death** |  |  |  |
| 2^nd^ tertile in strength of state social distancing measures^c^ | -0.13 | 0.88 (0.79-0.98) | 0.017 |
| 3^rd^ tertile in strength of state social distancing measures^c^ | -0.17 | 0.84 (0.75-0.94) | 0.003 |
| **Part 2: count of deaths conditional on ≥1 death** |  |  |  |
| 2^nd^ tertile in strength of state social distancing measures^c^ | 0.03 | 1.03 (0.95-1.11) | 0.464 |
| 3^rd^ tertile in strength of state social distancing measures^c^ | 0.07 | 1.07 (0.99-1.16) | 0.093 |
|  |  |  |  |
| **Two-part model for non-COVID-19 deaths among residents** |  |  |  |
| **Part 1: likelihood of ≥1 death** |  |  |  |
| 2^nd^ tertile in strength of state social distancing measures^c^ | -0.09 | 0.87 (0.86-0.97) | 0.004 |
| 3^rd^ tertile in strength of state social distancing measures^c^ | -0.02 | 0.98 (0.92-1.04) | 0.470 |
| **Part 2: count of deaths conditional on ≥1 death** |  |  |  |
| 2^nd^ tertile in strength of state social distancing measures^c^ | 0.07 | 1.07 (0.95-1.21) | 0.255 |
| 3^rd^ tertile in strength of state social distancing measures^c^ | 0.15 | 1.16 (1.02-1.31) | 0.020 |

^a^ Based on two-part models for confirmed cases and deaths separately that adjusted for nursing home, county, and state covariates, time trend, and the clustering of repeated observations of nursing homes.

^b^ ORs are reported for part 1 of the two-part models and IRR are reported for part 2 of the two-part models.

^c^ Compared to the 1^st^ tertile group.

OR=odds ratio; IRR=incidence rate ratio; 95% CI=95% confidence interval.
